# Boosting Cortical Activity at Beta-Band Frequencies Slows Movement in Humans

**DOI:** 10.1016/j.cub.2009.07.074

**Published:** 2009-10-13

**Authors:** Alek Pogosyan, Louise Doyle Gaynor, Alexandre Eusebio, Peter Brown

**Affiliations:** 1 Sobell Department of Motor Neuroscience and Movement Disorders, Institute of Neurology, University College London, London WC1N 3BG, UK

**Keywords:** SYSNEURO

## Abstract

Neurons have a striking tendency to engage in oscillatory activities. One important type of oscillatory activity prevalent in the motor system occurs in the beta frequency band, at about 20 Hz. It is manifest during the maintenance of tonic contractions and is suppressed prior to and during voluntary movement [1–7]. This and other correlative evidence suggests that beta activity might promote tonic contraction, while impairing motor processing related to new movements [3, 8, 9]. Hence, bursts of beta activity in the cortex are associated with a strengthening of the motor effects of sensory feedback during tonic contraction and with reductions in the velocity of voluntary movements [9–11]. Moreover, beta activity is increased when movement has to be resisted or voluntarily suppressed [7, 12, 13]. Here we use imperceptible transcranial alternating-current stimulation to entrain cortical activity at 20 Hz in healthy subjects and show that this slows voluntary movement. The present findings are the first direct evidence of causality between any physiological oscillatory brain activity and concurrent motor behavior in the healthy human and help explain how the exaggerated beta activity found in Parkinson's disease can lead to motor slowing in this illness [14].

## Results and Discussion

Our aim was to establish whether beta activity in the human motor system fashions motor behavior or is merely associated with it as an epiphenomenon. To this end, we applied an external source of beta oscillations to the motor cortex by using sinusoidal trancranial alternating-current stimulation (tACS) at 20 Hz while subjects performed a visuomotor task. This form of stimulation has been shown to entrain the discharge of spontaneously active neurons in slice preparations [15, 16] and to entrain cortical oscillations in humans in a safe and painless manner [17, 18]. We hypothesized that tACS in the beta range would help synchronize local cortical activity in this frequency band and thereby impair the execution of new movements, providing evidence that beta activity is mechanistically important in motor behavior.

**Figure 1:**
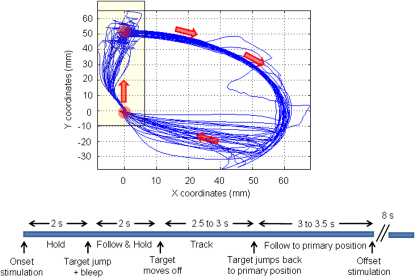
Task Upper panel shows a schematic of the trajectory of the target (red circle and arrows) on the monitor screen and 25 superimposed trials of movement of the cursor controlled by the joystick (blue traces). Only those trials performed during 20 Hz stimulation are shown. The coordinates are as measured on the screen, with 0 being the primary position in the center of the screen. A 5 cm movement of the cursor on the screen necessitated 4 degrees of movement of the joystick. Below the panel is the timeline of events. The principle behavioral event of interest was the rapid movement of the joystick to bring the cursor to the new position of the target after its jump (see yellow-shaded box in upper panel). Neither 20 Hz nor 5 Hz stimulation affected the reaction time or mean velocity of tracking of the continuous movement of the target later in the trial (Table S2). In stimulation trials, the initial target jump occurred 2 s after the onset of stimulation.

**Figure 2:**
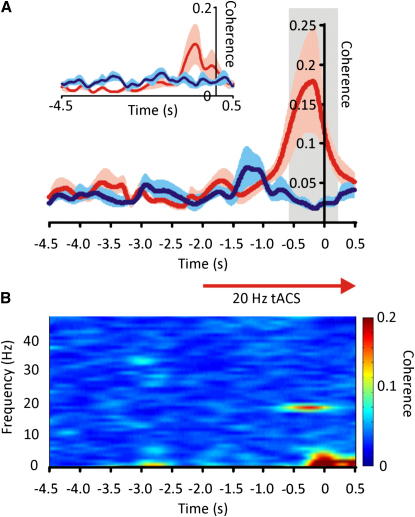
Time-Evolving Coherence between Scalp-Recorded Activity and Rectified EMG with and without 20 Hz Stimulation Scalp activity was recorded from a bipolar pair of electrodes placed medial to the stimulating sponge electrode and EMG from the first dorsal interosseous muscle of the hand holding the joystick. (A) Time-evolving coherence at 20 Hz during stimulation of the contralateral motor cortex at 20 Hz (red line) and without stimulation (blue line). Color-coded shaded areas represent ± standard error of the mean (SEM). There was a delayed increase in coherence during stimulation. Boxed area indicates the period over which coherence differed with and without stimulation (serial paired t tests over time, p < 0.05). Time is indicated with respect to jump in target spot. Smaller inset is time-evolving coherence between scalp-recorded activity and rectified EMG with time now indicated with respect to the onset of the movement made to catch up with the target jump. (B) Time-evolving spectrum of coherence during stimulation of the contralateral motor cortex at 20 Hz showing that stimulation did not result in any other spectral change. Arrow denoting timing of stimulation applies to (A) and (B). Data in (A) and (B) averaged over all subjects. Time-evolving coherence was determined with overlapping 1 s blocks centered on the timing used to denote each block.

Fourteen healthy subjects held a joystick in their dominant hand, which controlled the position of a spot on the center of a computer screen. Their task was to keep the spot within a target circle that jumped 5 cm vertically every 18 s in association with an auditory cue (Supplemental Experimental Procedures and Results). Subjects were requested to move the spot to the center of the new position of the target circle as quickly as possible and then hold this position until the target circle moved off in a continuous sweep (Figure 1). Thereafter, subjects were asked to track the movement of the target until it jumped back to its starting position, when they too had to return the joystick-controlled cursor to this position. One in two randomly selected trials occurred during stimulation. Stimulation began 2 s before the first target jump in each trial and lasted 10 s. It consisted of tACS applied at 20 Hz through a sponge electrode placed over the motor cortex contralateral to the active hand. The other electrode was positioned over the ipsilateral side of the neck. This electrode was placed away from the brain and had three times the surface area of the cortical electrode so as to reduce current density and limit any stimulation effects under this electrode. Stimulation intensity (0.58 ± 0.04 mA) was kept 0.1 to 0.2 mA below the threshold for visual flashes or skin sensation. Even so, stimulation was able to reorganize the temporal patterning of activity in the motor cortex prior to the first target jump, as evidenced by increased coherence between scalp-recorded activity and electromyographic activity (EMG) in the first dorsal interosseous muscle of the hand at 20 Hz during stimulation (Figure 2). Such coherence was not the product of volume conduction of the stimulation waveform. First, stimulation had to be sustained for 1.12 ± 0.23 s before entrainment of cortical output was fully established. The latter was determined from the time point at which coherence between scalp-recorded activity and EMG exceeded 95% confidence limits (CL). Second, phase estimates (Supplemental Experimental Procedures and Results and Figure S1) confirmed that scalp-recorded activity led EMG by 41.6 ± 6.9 ms, consistent with the synchronization of muscle activity by cortical outflow. Importantly, stimulation did not change the mean level of rectified EMG in the hand holding the joystick (29.9 ± 8.1 uV and 29.4 ± 7.6 uV before and after stimulation), but rather the precise timing of composite muscle action potentials as driven by the motor cortex. Note that the elevation in coherence with EMG, although delayed in onset, remained significant until after the first target jump (Figure 2A) but was beginning to fall off by the time of onset of the movement made to catch up with the target (Figure 2A, inset). Spontaneous cortical activity in this frequency band is similarly suppressed around the time of voluntary movement [1–7]. This suggests that the effects of tACS are affected by the state of the underlying cortical circuits in much the same way as spontaneous oscillations of similar frequency. A similar interaction between tACS and the natural frequency of underlying cortical circuits has also been noted in the visual cortex [18].

**Figure 3:**
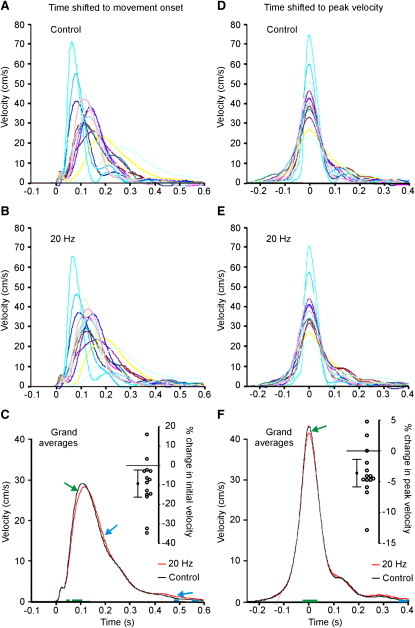
The Velocity Profiles of Trials Performed with and without 20 Hz Stimulation In (A) and (B), the mean velocities from 14 individuals have been realigned to response onset, and in (C) these have been further averaged across individuals. In (D) and (E), the velocity profiles in each trial have been realigned to peak velocity and then averaged for each of the 14 subjects, and in (F) these have been further averaged across subjects. The mean ± 2 SEM and the spread of individual % changes in velocity upon tACS at 20 Hz are shown to the right of (C) and (F). Green and blue arrows in (C) and (F) draw attention to periods in which mean velocity is slower and faster during stimulation. The bars of corresponding color along the x axes in (C) and (F) highlight periods of significant difference (serial two-tailed paired t tests p < 0.05).

Reaction time in the task was unaffected by stimulation (255 ± 13 ms and 249 ± 14 ms with and without 20 Hz stimulation, t_[13]_ = 1.492, p = 0.160), consistent with the imperceptible nature of stimulation. Nevertheless, reaction time varied between subjects, so we realigned individual averages according to response onset before calculating velocity from the initial position to the relocated target (Supplemental Experimental Procedures and Results). Figure 3 shows the mean velocity traces for each subject realigned to the onset of movement (Figures 3A–3C). It reveals a small but significant initial slowing of velocity during stimulation at 20 Hz (green arrow in Figure 3C), followed by a slowed deceleration (blue arrows in Figure 3C), so that the target was eventually reached. Averaging of the initial velocity from 40 to 100 ms after movement onset confirmed that it was reduced by stimulation (17.8 ± 3.6 cm/s and 19.6 ± 4.0 cm/s with and without 20 Hz stimulation, t_[13]_ = −2.717, p = 0.018, paired t test; 9.3% ± 3.5% reduction with 20 Hz stimulation, t_[13]_ = −2.664, p = 0.019, single-sample t test). The right-hand panels of Figures 3D–3F show the velocity traces realigned to peak velocity on each trial and then averaged for each subject. There is a small but significant additional slowing of peak velocity during stimulation at 20 Hz (41.5 ± 3.3 cm/s and 43.1 ± 3.5 cm/s with and without 20 Hz stimulation, t_[13]_ = −3.347, p = 0.005; 3.6% ± 1.1% reduction with 20 Hz stimulation, t_[13]_ = −3.212, p = 0.007; green arrow in Figure 3F).

**Figure 4:**
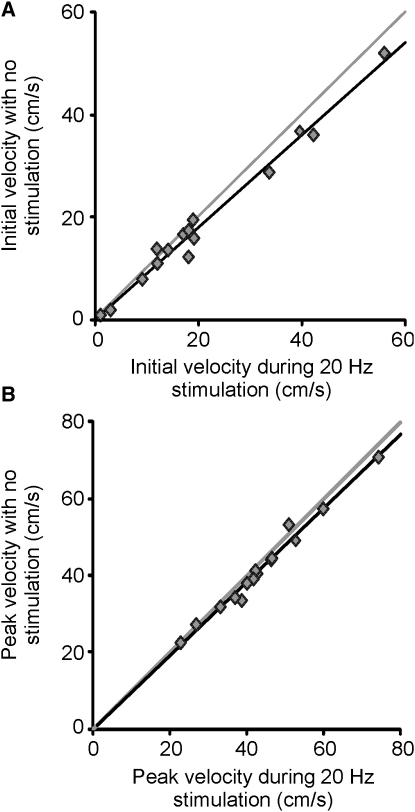
The Dependency of the Slowing of Velocity during Stimulation at 20 Hz upon Baseline Performance (A) Initial velocity (average over 40–100 ms after response onset). (B) Peak velocity. Gray lines mark gradients of 1. Note that the majority of points fall below the gray lines, indicating that stimulation slows velocity. In addition, linear regression lines (black) have gradients of < 1, suggesting that the slowing of velocity with stimulation at 20 Hz is greater in subjects making more rapid movements. See Results and Discussion for statistics.

However, the above group analyses obscure any dependence of the effect of stimulation at 20 Hz on baseline performance. Such dependence would be important as it would provide evidence that stimulation was interacting with motor execution and hence having an effect upon the motor cortex directly under the stimulation electrode. Linear regression analysis between initial velocity with 20 Hz stimulation and initial velocity without stimulation confirmed this key interaction, demonstrated by a gradient that was less than unity (r^2^ = 0.980, F_[1,12]_ = 601.6, p < 0.001; mean gradient 0.897 with 95% CL 0.817 to 0.977, t = 24.5, p < 0.001; Figure 4A). A gradient of 1 would have been expected had there been no interaction between the effect of stimulation at 20 Hz and baseline performance. A similar trend was seen between peak velocity with 20 Hz stimulation and peak velocity without stimulation (r^2^ = 0.981, F_[1,12]_ = 630.4, p < 0.001; mean gradient 0.956 with 95% CL 0.873 to 1.039, t = 25.1, p < 0.001; Figure 4B). A similar dependency of the slowing of velocity during stimulation at 20 Hz upon baseline performance was also evident within subjects (Supplemental Results).

The above suggests that stimulation at 20 Hz slows initial and peak velocity compared to no stimulation, and does so more the faster the original velocity. However, it does not provide any evidence that the effect of stimulation was frequency selective. Accordingly, we repeated the experiment with similar stimulation intensities on another occasion, but contrasting 5 Hz stimulation with no stimulation in 11 of the original subjects (Supplemental Experimental Procedures and Results). The very similar stimulation intensities used for 5 Hz (0.58 ± 0.04 mA) and 20 Hz (0.57 ± 0.04 mA; t_[10]_ = 0.199, p = 0.846) sinusoidal stimulation meant that the integrated negative- and positive-current profiles and root mean square power of the two stimulation trains were no different. The waveforms only differed in frequency, and time-evolving spectra of the coherence between scalp-recorded activity and EMG confirmed that stimulation at 5 Hz did not elevate coherence at 20 Hz (Figure S2). Under these circumstances, there was no effect of stimulation on reaction time (273 ± 23 ms and 278 ± 23 ms with and without 5 Hz stimulation, t_[10]_ = −1.179, p = 0.266), initial velocity (21.8 ± 5.5 cm/s and 21.8 ± 5.5 cm/s with and without 5 Hz stimulation, t_[10]_ = −0.082, p = 0.936; 0.9% ± 3.7% reduction with 5 Hz stimulation, t_[10]_ = −0.242, p = 0.813; Figures S3A–S3C), or peak velocity (44.5 ± 5.8 cm/s and 44.9 ± 5.7 cm/s with and without 5 Hz stimulation, t_[10]_ = −0.475, p = 0.645; 1.1% ± 2.1% reduction with 5 Hz stimulation, t_[10]_ = −0.511, p = 0.620; Figures S3D–S3F). In line with this, linear regression analysis between initial velocity with 5 Hz stimulation and initial velocity without 5 Hz stimulation failed to show any interaction between these variables (r^2^ = 0.993, F_[1,9]_ = 1193.8, p < 0.001; mean gradient 0.997 with 95% CL 0.932 to 1.063, t = 34.6, p < 0.001; Figure S4A); neither was there an interaction between peak velocity with 5 Hz stimulation and peak velocity without 5 Hz (r^2^ = 0.982, F_[1,9]_ = 501.9, p < 0.001; mean gradient 1.001 with 95% CL 0.907 to 1.110, t = 22.4, p < 0.001; Figure S4B). Most importantly, however, a linear mixed model of initial velocities with 5 and 20 Hz stimulation used initial velocity without stimulation as a covariate in the 11 subjects tested with both frequencies and demonstrated an interaction between stimulation frequency and baseline performance (F_[1,9.5]_ = 5.847, p = 0.037). Similarly, a linear mixed model of peak velocities with 5 and 20 Hz stimulation used peak velocity without stimulation as a covariate and demonstrated an interaction between stimulation frequency and baseline performance (F_[1,9.6]_ = 5.993, p = 0.035). Both interactions were due to the dependency of the effects of 20 Hz, but not 5 Hz, stimulation on baseline performance (compare Figure 4 and Figure S4). In summary, the results indicated that the reduction in the velocity of voluntary movement with stimulation at 20 Hz depended on baseline performance, whereas stimulation at 5 Hz was ineffective, regardless of baseline performance. Neither 20 Hz nor 5 Hz tACS affected the reaction time, error, or mean velocity of tracking of the continuous movement of the target later in the trial (Supplemental Experimental Procedures and Results).

It seems likely that the slowing of voluntary movement by stimulation at 20 Hz involves a specific effect upon motor processing insofar as stimulation could not be perceived and the effect of stimulation was dependent on frequency, and, perhaps most importantly, on the velocity with which the task was performed at baseline. Furthermore, the slowing of movement was probably caused through manipulation of activity in the primary motor cortex, the site of the maximal current density. The nature of the effect is in line with the motor cortex's pivotal role in the coding of force [19] and also with the observed slowing of voluntary movement (without change in reaction time) in healthy subjects when action is triggered by spontaneous bursts of beta activity in the motor cortex [9].

The present results show that an intervention capable of artificially synchronizing cortical activity in the beta frequency band slows voluntary movement but do not in themselves explain how increased beta synchrony affects motor processing. There is increasing evidence that beta oscillations underpin the integration of sensorimotor processes important in the maintenance of tonic motor output [1]. This may be achieved through superior communication [20, 21] and, possibly, through the promotion of short-term plasticity, given that the behavioral associations of beta activity often outlive beta bursts by a few 100 ms [9, 10].

In summary, the current findings provide the first interventional evidence of a causal rather than associational link between increased beta synchrony and the slowing of voluntary movement in otherwise healthy subjects. It has previously been shown that transcranial application of oscillating potentials of very low frequency (0.75 Hz) during non-rapid-eye-movement sleep enhances the retention of hippocampus-dependent declarative memories when tested the next morning in healthy humans [17]. The present findings are the first direct evidence of causality between any oscillatory activity and concurrent motor behavior in the healthy human. The importance of the findings is heightened by the exaggeration of beta synchrony at basal ganglia and cortical levels evident in Parkinson's disease, a condition typified by the impairment of voluntary movement [14].

## Supplemental Data

Document S1. Supplemental Experimental Procedures, Results, Two Tables, Four Figures
