# Supplementary material for: Boosting Cortical Activity at Beta-Band Frequencies Slows Movement in Humans
Source: Curr Biol. 2009 Oct 13;19(19):1637–41. doi: 10.1016/j.cub.2009.07.074 (PMC2791174; doi:10.1016/j.cub.2009.07.074)
Supplement: Document S1. Supplemental Experimental Procedures, Results, Two Tables, Four Figures [file mmc1.pdf]

Current Biology, Volume 19

Supplemental Data

Boosting Cortical Activity at Beta-Band Frequencies Slows Movement in Humans

Alek Pogosyan, Louise Doyle Gaynor, Alexandre Eusebio, and Peter Brown

Supplemental Experimental Procedures

Subjects

Fourteen AC-stimulation naive subjects (1 female, all right-handed) gave informed consent to take part in the study, which was approved by the ethics committee of University College London. All experiments conformed to the Declaration of Helsinki. The experiment consisted of two separate sessions involving stimulation at 20 Hz or 5Hz and control trials without stimulation. Sessions were conducted 72 ± 5 days apart in randomized order.

Stimulation

tACS was applied through conductive-rubber electrodes enclosed in saline-soaked sponges using a commercial battery-driven constant-current stimulator (World Precision Instruments, Stevenage, UK) with a maximum output of 10 mA. The stimulation electrode (4.5 cm x 3.2 cm) was placed over the left motor hotspot, determined with transcranial magnetic stimulation of the cortex [1], while the bigger reference electrode (7.6 cm x 5.5 cm) was positioned on the contralateral side of the neck. The electrodes were held in place with velcro

bands. The stimulating current was delivered at intensities up to 0.7 mA for periods of 10 s separated by periods of no stimulation for at least 8 s. This arrangement of electrodes, together with the subthreshold intensity of stimulation eliminated any visual flashes, muscle responses and discomfort upon stimulation so that subjects remained blinded to the intervention. The latter was confirmed by asking subjects to make forced guesses as to whether or not stimulation was delivered during the initial trials of the experiment. The experiment comprised two sessions of three blocks in which 10 periods of stimulation were randomly interspersed with 10 periods of no stimulation with rest periods of 1-2 minutes between each block. Experiments involved equal numbers of stimulation and non-stimulation trials. Stimulation was at either 20 Hz or 5 Hz, with 72 ± 5 days between sessions of different frequency.

Task

Subjects performed a reaching task with and without tACS over the motor cortex. This involved fixating on a spot of 5 mm diameter inside a larger target circle of 10 mm diameter at the centre of a portable PC screen until the target reappeared at a point 5 cm (equivalent to a shift in visual angle of 3.2 deg and of the joystick of 4.0 deg) above the initial location following an auditory cue that was delivered 2 s after the onset of stimulation. Subjects were required to reposition the small spot within the target circle as fast as possible by moving a custom-made joystick (9 cm shaft) held in the right hand. They then had to hold the small spot in position in the target circle for 2 s before tracking the target as it moved

continuously for 2.5-3 s. Finally the target jumped back to its original location in the centre of the monitor screen, when subjects also returned the joy-stick controlled cursor back to this position.

Recording

During the task, EEG activity and the stimulus train were recorded from a bipolar pair of electrodes over the scalp medial to the stimulating sponge electrode. EMG activity was also recorded bipolarly from the first dorsal interosseus of the (contralateral) right hand with one electrode over the muscle belly and the second over the first metacarpo-phallangeal joint. EEG, EMG and joystick signals were sampled at 2048Hz, amplified (x 20) and band-pass filtered at 1 – 250Hz using a Porti32 amplifier (Twente Medical Systems International, Oldenzaal, The Netherlands).

Analysis

Signals were edited using custom-made software (EditEEG©, developed by A.P.) and sections deleted in which scalp recorded activity was dominated by muscle arfetact. Recordings were then imported into Spike© version 6.10 (Cambridge Electronic Design, Cambridge, UK) using a custom-made script and trials in which responses had a reaction time of under 100 ms or trials in which trajectories departed from within a region bounded by ± (3 x standard deviations) of the group data were deleted. There was no difference between the percentage

of stimulated and non stimulated trials rejected (Supplementary Table 1). Further analysis was performed in MATLAB.

Scalp recorded activity and EMG spectra were estimated for the initial hold phase, prior to the first target jump, using the discrete Fourier transform [2, 3]. Scalp recorded activity included any stimulation signal and underlying EEG, and no attempt was made to distinguish the two as driven cortical activity would have been phase locked to the stimulus and hence inseparable. We worked on the assumption that what was recorded at the scalp could be taken to index what was going on at the cortex. This is borne out by the elevated coherence between the scalp recorded activity and muscle and by the corresponding phase estimates which indicate cortical driving of muscle activity.

We did not perform spectral analysis of the period just after the first target jump as the hold phase that followed cursor movement was shorter and would have been confounded by post-movement beta rebound. The latter is an important issue as the cursor movement to catch up with the position of the target differed according to whether or not 20 Hz stimulation was applied, so that the post-movement beta rebound might have differed because of a concurrent effect of stimulation or because of the effect of stimulation performed earlier during the course of the movement. Scalp recorded signals were assumed to be realizations of stationary, zero-mean time series and to satisfy a mixing condition, whereby sample values widely separated in time were independent. Records were divided

into a number of sections of equal duration with a block size of 1 s (2048 data points), affording a frequency resolution of 1 Hz. Spectra were estimated by averaging across sections and a Hanning window filter used. Blocks were shifted by 10 ms and averaged again until the whole record length had been analysed using a modified Spike2 script. Coherence between scalp recorded activity and rectified FDI EMG of the hand holding the joy-stick was estimated for data re-aligned to the onset of the imperative signal (target jump and auditory cue). Coherence at 20 Hz was normally distributed and therefore not transformed before separately averaging data from stimulation and non-stimulation trials across the group (Figure 2). Coherence at 20 Hz was also considered individually, and exceeded 95 % confidence levels [2] for at least 10 consecutive blocks of the moving window in eight subjects during stimulation at 20 Hz. Phase was analysed in these subjects by linear regression of estimates over 19 Hz, 20 Hz and 21 Hz [3]. Five subjects had correlation coefficients > 0.95, allowing reliable estimates to be computed of the temporal difference between scalp and muscle signals (Figure S1). Finally, the timing of the occurrence of coherence at 20 Hz was estimated by realigning the data to the time of onset of stimulation. The time point at which coherence between scalp recorded activity and EMG exceeded 95% confidence limits was measured in each subject, providing this did not occur after presentation of the imperative cue. Time estimates were available in 8 subjects and averaged.

We also checked that stimulation at 20 Hz did not affect the level of EMG activity in the hand holding the joystick prior to onset of the imperative cue. To this end we contrasted the mean rectified EMG in the first dorsal interosseous over the 1 s before with that over the 1 s after onset of stimulation.

Reaction time varied between subjects. Accordingly we re-aligned averages of the velocity profiles in each subject according to response onset before considering the data at the group level. Velocity was calculated by differentiation of the spatial coordinates of the joystick. Response onset was defined as the time point at which spot velocity exceeded 2 % of maximal velocity. Variability in kinematic profile between individuals was offset by always performing paired comparisons of stimulation and non-stimulation within subjects. Nevertheless, the results of re-alignment to response onset could potentially be affected by the criteria used to determine response onset. We therefore also analysed an additional kinematic measure that could be independently defined; the peak velocity.

Kolmogorov-Smirnov tests confirmed that data were normally distributed. Means ± standard error of the means are specified. Initial and peak velocities were contrasted between stimulation conditions using a linear mixed model and two-tailed paired t-tests ($p < 0.05$; SPSS Inc., Chicago).

Supplemental Results

The dependency of the slowing of velocity during stimulation at 20 Hz upon baseline performance was examined within subjects. As individual trials with and without 20 Hz stimulation were not paired, we split the data in each individual subject into tertials (thirds) ordered with respect to mean or peak velocity. Thus the tertial with the slowest peak velocity during stimulation was paired with that with the slowest peak velocity without stimulation in each subject. We then performed a linear mixed model of peak velocities in the different tertials. Analysed in this way, the results did hold at an individual level for peak velocity, as the interaction between tertial order and baseline peak velocity was significant ($F_{[27.7]} = 3.840$, $p = 0.034$). There was also a modest trend for a similar interaction between tertial order and baseline average velocity ($F_{[27.1]} = 1.910$, $p = 0.167$).

Neither 20 Hz nor 5 Hz tACS affected the reaction time or mean velocity of tracking of the continuous movement of the target later in the trial (Supplementary Table 2).

Supplemental References

1. Münchau, A., Bloem, B.R., Irlbacher, K., Trimble, M.R., and Rothwell, J.C. (2001). Functional connectivity of human premotor and motor cortex explored with repetitive transcranial magnetic stimulation. J. Neurosci. 22, 554-561.

2. Halliday, D.M., Rosenberg, J.R., Amjad, A.M., Breeze, P., Conway, B.A., and Farmer, S.F. (1995). A framework for the analysis of mixed time series/point process data--theory and application to the study of physiological tremor, single motor unit discharges and electromyograms. Prog. Biophys. Mol. Biol. 64, 237-278.

3. Grosse, P., Cassidy, M., and Brown, P. (2002). EEG-EMG, MEG-EMG and EMG-EMG frequency analysis: physiological principles and clinical applications. Clin. Neurophysiol. 113, 1523-1531.

4. Gilbertson, T., Lalo, E., Doyle, L., Di Lazzaro, V., Cioni, B., Brown, P. (2005). Existing motor state is favored at the expense of new movement during 13–35 Hz oscillatory synchrony in the human corticospinal system. J Neurosci 25, 7771-7779.

5. Williams, E.R., Baker, S.N. (2009). Renshaw cell recurrent inhibition improves physiological tremor by reducing corticomuscular coupling at 10 Hz. J Neurosci. 29, 6616-6624.

	**Stimulation**	**No stimulation**	**Significance**
20 Hz (n = 14)	10.0 ± 0.7	9.2 ± 0.6	P = 0.718
5 Hz (n=11)	10.3 ± 0.5	11.5 ± 0.4	P = 0.444

Table S1: Percentage trials rejected per condition. There was no difference between stimulation and non stimulation.

	**Stimulation**	**No stimulation**	**Significance**
**Delay to onset of tracking**			
20 Hz (n = 14)	257 ± 4 ms	258 ± 4 ms	P = 0.898
5 Hz (n=11)	249 ± 4 ms	249 ± 5 ms	P = 0.768
**Initial velocity to catch target up**			
20 Hz (n = 14)	60.7 ± 3.8 cm/s	60.2 ± 3.9 cm/s	P = 0.616
5 Hz (n=11)	57.7 ± 3.7 cm/s	56.5 ± 3.9 cm/s	P = 0.310
**Tracking velocity**			
20 Hz (n = 14)	35.1 ± 0.4 cm/s	35.3 ± 0.5 cm/s	P = 0.440
5 Hz (n=11)	35.3 ± 0.3 cm/s	35.6 ± 0.3 cm/s	P = 0.152

Table S2: Behavioural results from later tracking phase of task. Delay to onset of tracking is the time delay before the joy-stick controlled cursor is moved to catch-up with the moving target. Initial velocity to catch target up is the mean velocity over the 0.1 s after onset of cursor movement. Tracking velocity is the mean velocity over 0.1 s to 1.0 s after onset of cursor movement.

Figure S1. Example of coupling between tACS signal at 20 Hz and EMG in hand in one subject. [A] Raw data. [B] Coherence spectrum between scalp recorded tACS signal and rectified EMG from first dorsal interosseous muscle of contralateral hand during holding of the joystick prior to presentation of imperative cue. Thin horizontal line is 95% confidence limit. [C] Corresponding phase spectrum. Phase estimates from 3 contiguous bins with significant coherence from 19-21 Hz are shown in black. Linear regression of these estimates suggests that activity at scalp preceded EMG by ~30 ms ($r = 0.9998$, $p = 0.01$).

Figure S2. Time-evolving spectrum of coherence between scalp recorded activity and rectified EMG in first dorsal interosseous muscle in the hand during 5 Hz stimulation. Time indicated with respect to jump in target spot. Stimulation of the contralateral motor cortex at 5 Hz did not result in any increase in coherence at 20 Hz. However, although the current density used was the same as that used during stimulation at 20 Hz, there was also no coherence at 5 Hz. The latter is consistent with tuning (natural frequency) of cortical networks to around 20 Hz, at least during tonic contractions [4], or with filtering at the spinal level [5].

Figure S3. The velocity profiles of trials performed with and without 5 Hz stimulation. In A and B the mean velocities from 11 individuals have been re-aligned to response onset, and in C these have been further averaged across individuals. In D and E the velocity profiles in each trial have been re-aligned to

peak velocity and then averaged for each of the 11 subjects, and in F these have been further averaged across subjects. The mean ± 2 standard errors of the mean and the spread of individual % changes in velocity upon stimulation at 5 Hz are shown to the right of C and F. There was no change in either initial or peak velocity during stimulation at 5 Hz.

Figure S4. The dependency of the slowing of velocity during stimulation at 5 Hz upon baseline performance. A: initial velocity (average over 40-100 ms after response onset). B: peak velocity. Gray lines mark gradients of 1. Points are fairly evenly distributed around the gray lines and the linear regression lines (black) have gradients that are very close to 1. See Results for statistics.

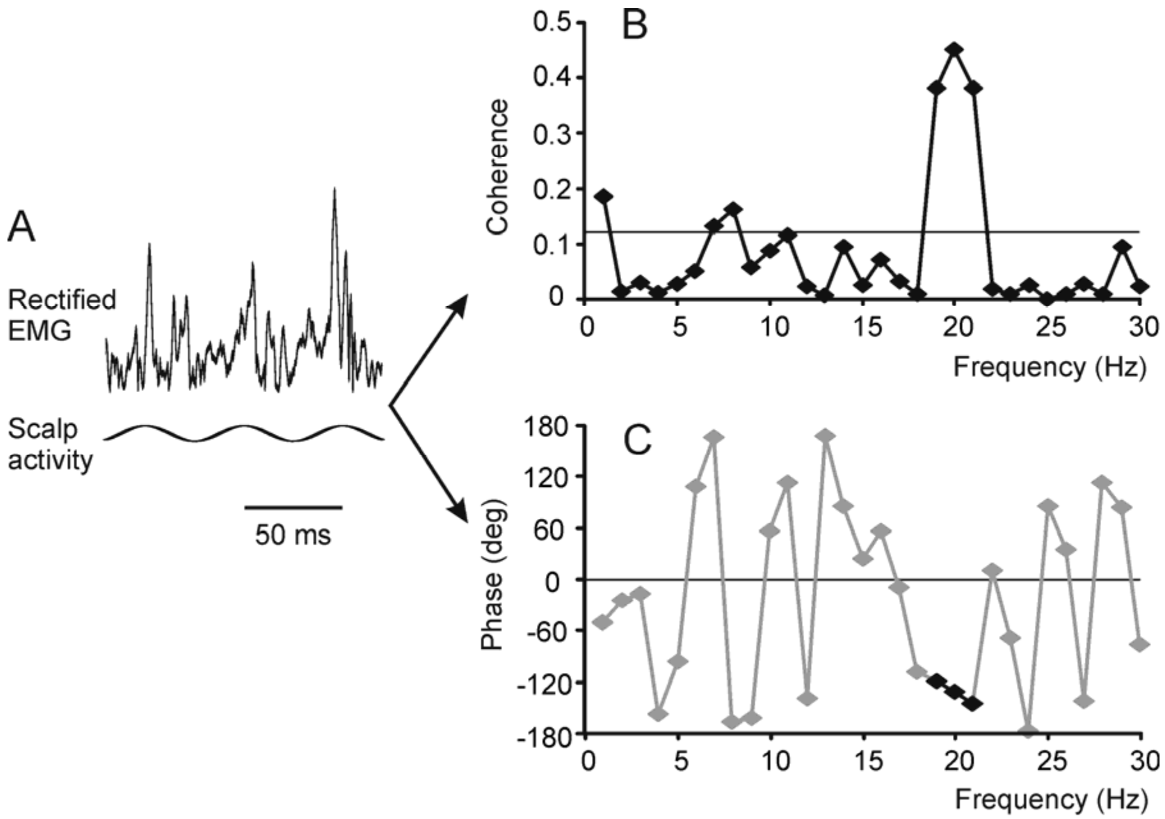
A
Rectified EMG
Scalp activity
50 ms
B
Coherence
0.5
0.4
0.3
0.2
0.1
0
0
5
10
15
20
25
30
Frequency (Hz)
C
Phase (deg)
180
120
60
0
-60
-120
-180
0
5
10
15
20
25
30
Frequency (Hz)

Figure S1

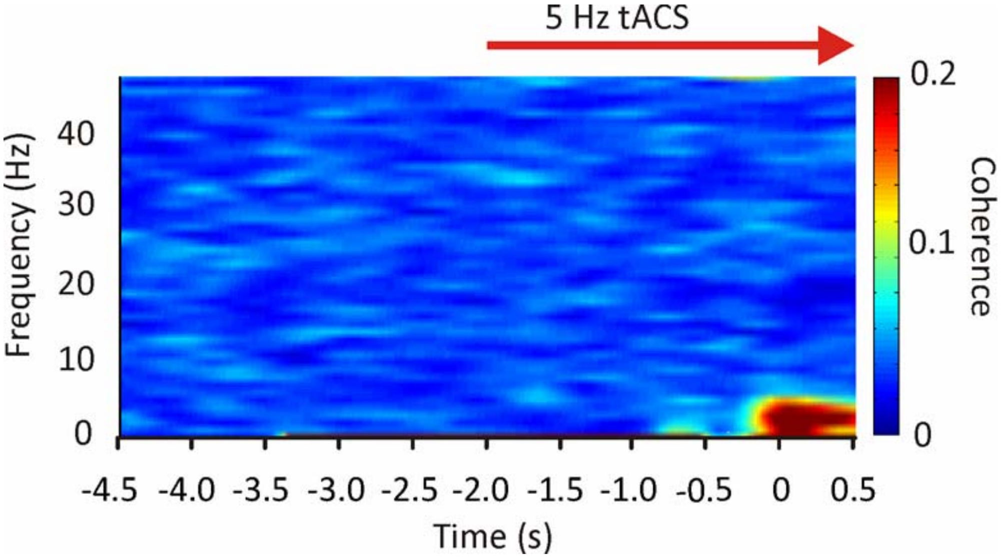
5 Hz tACS
Frequency (Hz)
40
30
20
10
0
-4.5 -4.0 -3.5 -3.0 -2.5 -2.0 -1.5 -1.0 -0.5 0 0.5
Time (s)
0.2
0.1
0
Coherence

Figure S2

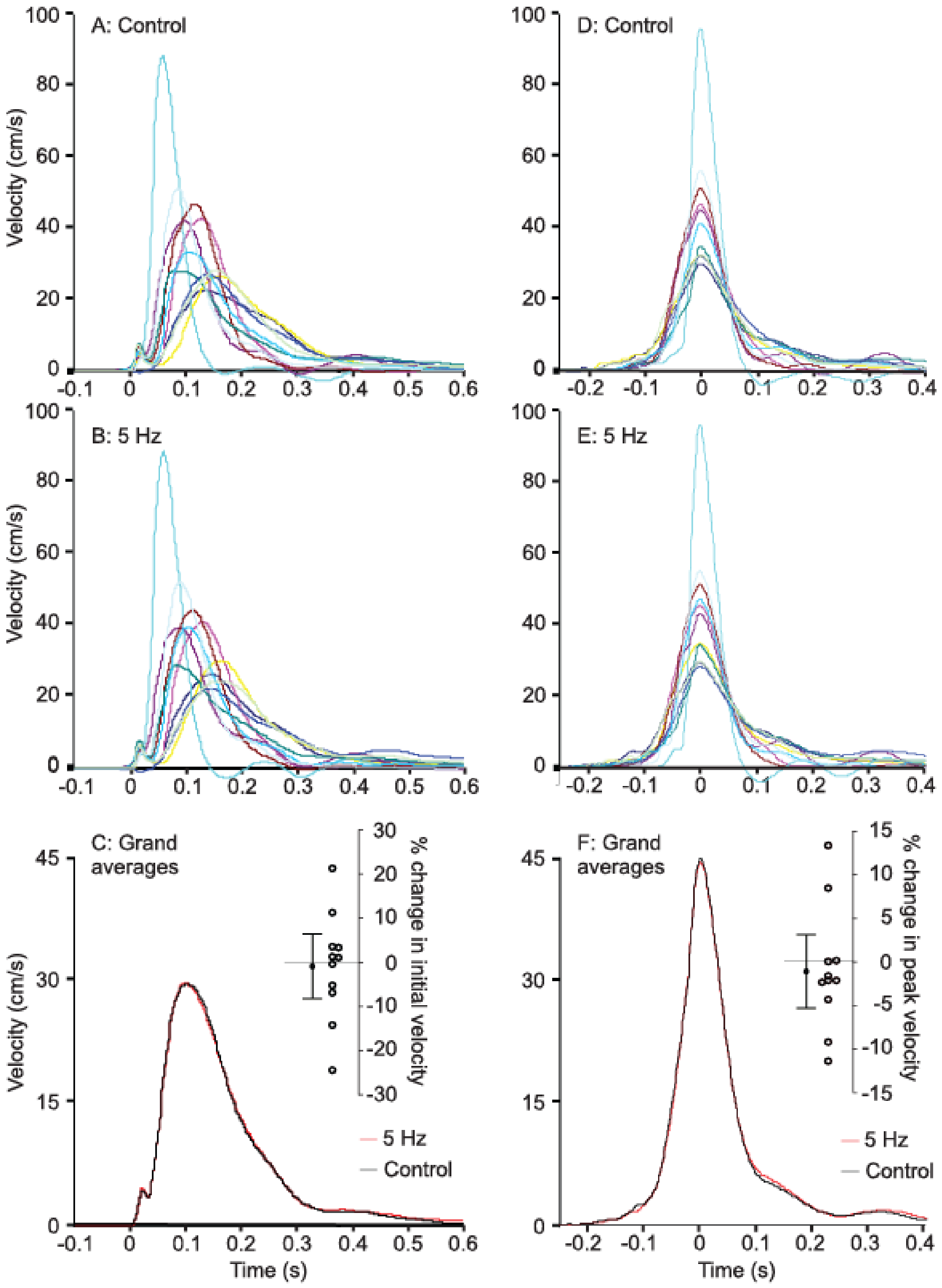
A: Control
B: 5 Hz
C: Grand averages
D: Control
E: 5 Hz
F: Grand averages
Velocity (cm/s)
Time (s)
% change in initial velocity
% change in peak velocity
5 Hz
Control

Figure S3

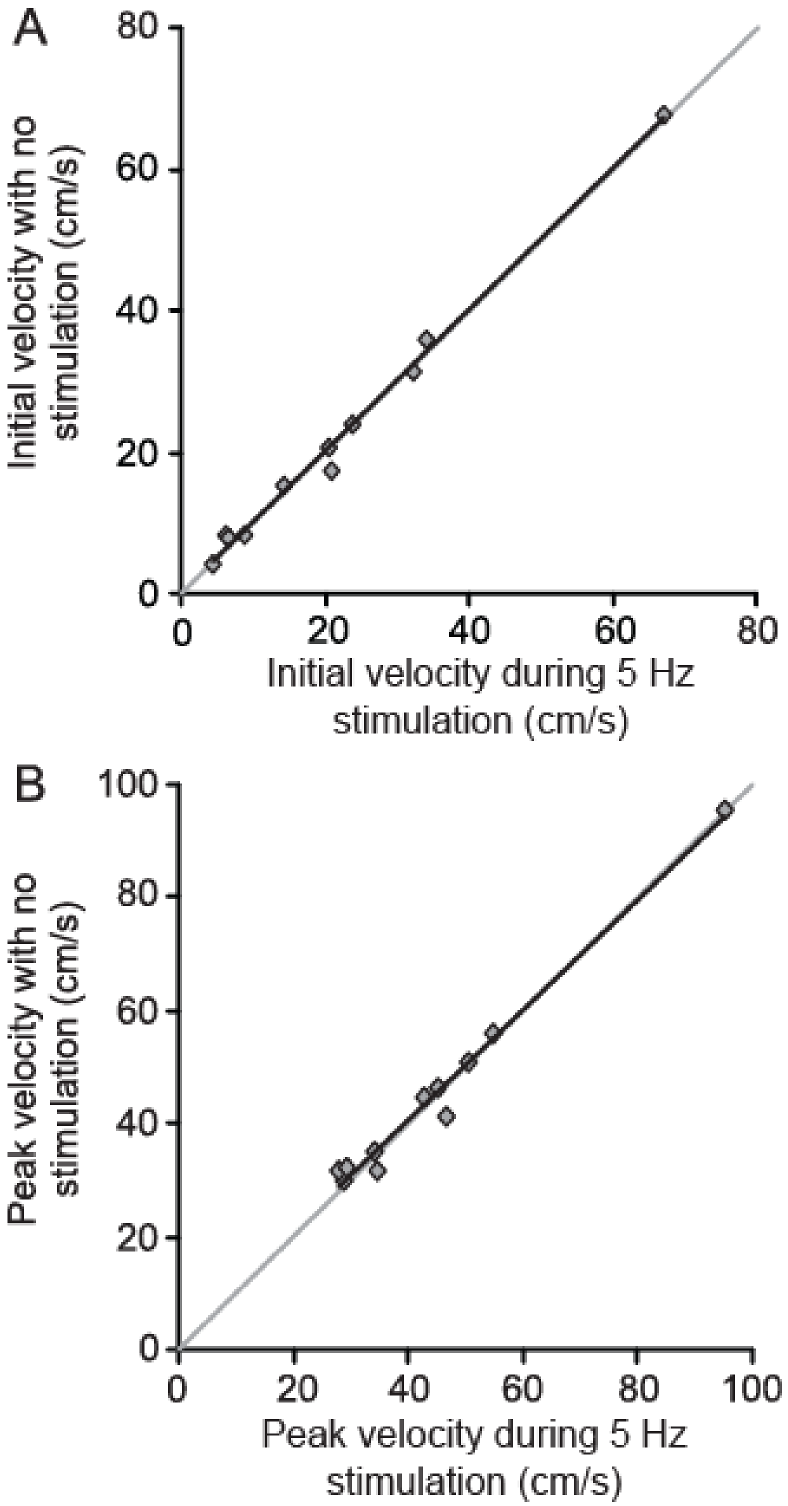
A
80
60
40
20
0
Initial velocity with no stimulation (cm/s)
0
20
40
60
80
Initial velocity during 5 Hz stimulation (cm/s)
B
100
80
60
40
20
0
Peak velocity with no stimulation (cm/s)
0
20
40
60
80
100
Peak velocity during 5 Hz stimulation (cm/s)

Figure S4
